# Supplementary material for: Multiple Head Rotations Result in Persistent Gait Alterations in Piglets
Source: Biomedicines. 2022 Nov 19;10(11):2976. doi: 10.3390/biomedicines10112976 (PMC9687234; doi:10.3390/biomedicines10112976)
Supplement: Supplementary file 1 [file biomedicines-10-02976-s001.zip › biomedicines-1993954-supplementary.pdf]

Table S1. Summary of daily group averages for each parameter. Values represent the calculated group average ± standard error.

	Pre-Injury			Day +1			Day +4			Day +7		
	Sham	Single	Multiple	Sham	Single	Multiple	Sham	Single	Multiple	Sham	Single	Multiple
Number of Stances	21.87 ±0.63	22.2± 0.67	16.7± 0.68	21.75± 1.27	19.3± 0.97	23.71 ±1.30	18.87 ±0.58	18.5± 0.65	20.71 ±0.91	18.12 ±0.74	17±0.45	18.28 ±0.56
Gait Time (s)	2.35± 0.17	2.74± 0.19	2.72± 0.24	2.31±0.25	3.39± 0.50	6.79± 2.21	1.96± 0.12	2.32± 0.17	2.54± 0.15	1.79± 0.12	2.10± 0.11	2.24± 0.25
Velocity (cm/s)	80.68 ±5.62	67.59 ±4.38	70.25 ±6.42	84.57± 10.1	60.16 ±5.66	42.21 ±10.2	97.37 ±5.62	79.99 ±4.77	76.35 ±4.42	102±7.83	88.5± 5.46	77.05 ±4.90
Cycle Time (s)	0.47± 0.03	0.54± 0.03	0.54± 0.04	0.47±0.03	0.64± 0.06	1.20± 0.34	0.44± 0.01	0.51± 0.02	0.57± 0.03	0.43± 0.01	0.50± 0.02	0.55± 0.039
Cycles Per Minute	133±8.29	114.6 ±6.7	116±7.5	134.3± 10.8	100±6.9	74±13.9	140.6 2±5.8	121.3 ±5.6	116.7 ±8.5	142.3 ±5.1	122.7 ±5.0	114.3 ±7.9
Stance Time (s)	0.23± 0.01	0.26± 0.00	0.31± 0.01	0.23±0.01	0.28± 0.01	0.67± 0.08	0.21± 0.01	0.26± 0.01	0.28± 0.01	0.21± 0.00	0.26± 0.00	0.30± 0.01
Stride Length (LF, cm)	36.99 ±0.34	35.74 ±0.30	36.63 ±0.65	37.56± 0.63	36.06 ±0.46	30.94 ±1.34	42.03 ±0.39	39.97 ±0.31	41.37 ±0.58	43.89 ±0.69	43.60 ±0.38	42.06 ±0.43
Stride Length (RF, cm)	36.81 ±0.28	35.86 ±0.32	37.10 ±0.68	37.50± 0.64	36.14 ±0.47	30.89 ±1.34	42.19 ±0.40	39.93 ±0.30	41.47 ±0.56	43.93 ±0.66	43.77 ±030	41.94 ±0.44
Stride Length (LH, cm)	35.23 ±0.37	32.35 ±0.47	34.11 ±0.90	35.74± 0.86	32.15 ±0.65	28.76 ±1.41	42.20 ±0.48	36.76 ±0.39	38.64 ±0.50	42.09 ±0.76	40.35 ±0.53	40.94 ±0.50
Stride Length (RH, cm)	34.68 ±0.41	32.13 ±0.45	34.71 ±0.90	35.58± 0.89	32.36 ±0.60	27.66 ±1.64	41.59 ±0.55	36.72 ±0.41	38.89 ±0.49	42.89 ±0.74	41.29 ±0.49	41.07 ±0.46

Table S2. Comparison between pediatric studies and single and multiple RNR injury models.

	**Pediatric Patients**			**Piglets (vs. SHAM)**	
	Paper	*TBI Children vs. Healthy Controls*	*Time Post-injury*	*SINGLE*	*MULTIPLE*
Gait Velocity **% Decrease**	Katz-Leurer et al. 2008 [59]	(27.74 %)	3-12 months	**(29 %)** (1-day post)	**(50 %)** (1-day post)
	Kuhtz-Buschbeck et al. 2003 [9]	(20 %)	12.1 months		
	Katz-Leurer et al. 2011 [10]	(50 %)	3.5 years		
	Kuhtz-Buschbeck et al. 2003 [7]	(23 %)	2.8 months		
		(7.05 %)	7.8 months		
	Beretta et al. 2009 [66]	(46.15 %)	1.4 months	**(18 %)** (4-day post)	**(22 %)** (4-day post)
		(38.46%)	5.5 months		
	Abdul-Rahman et al. 2021 [65]	(5.84 %) (Single task)	2.43 years		
		(12.45 %) (Concurrent motor task)			
		(11.84 %) (Concurrent cognitive task)			
	Howell et al. 2017 [67] (1 concussion)	(1.72 %) (Single task)	10.7 days		
		(3.30 %)			

		(Double task)			
	Howell et al. 2017 [67] (⩾ 2 concussions)	(7.76 %) (Single task)	8.6 days	**(13 %)** (7-day post)	**(24 %)** (7-day post)
		(12.08 %) (Double task)			
	Berkner et al. 2017 [13]	(6.78 %) (Single task)	9.5 days		
		(8.70 %) (Double task)			
	Katz-Leurer et al. 2011 [58]	(7.69 %) (Usual walking)	3.5 years		
		(38.46 %) (Dual-task numbers)			
		(36.36 %) (Dual-task sounds)			
Cadence **% Decrease**	Kuhtz-Buschbeck et al. 2003 [9]	(6.88 %)	12.1 months	**(26 %)** (1-day post)	**(45 %)** (1-day post)
	Kuhtz-Buschbeck et al. 2003 [7]	(13.25 %)	2.8 months		
		(5.56 %)	7.8 months		
	Abdul-Rahman et al. 2021 [65]	(5.3 %) (Single task)	2.43 years		
		(9.14 %) (Concurrent motor task)			
		(10.46 %) (Concurrent cognitive task)		**(14 %)** (4-day post)	**(17 %)** (4-day post)
	Howell et al. 2017 [67] (1 concussion)	(0.4 %) (Single task)	10.7 days		
		(1.40 %) (Double task)			
	Howell et al. 2017 [67] (⩾ 2 concussions)	0.089 % (Single task)	8.6 days		
		(3.81 %) (Double task)			
	Berkner et al. 2017 [13]	0.089 % (Single task)	9.5 days		
		(1.31 %) (Double task)		**(14 %)** (7-day post)	**(20 %)** (7-day post)
	Abdul-Rahman et al. 2018 [68]	(5.69 %) (Single task)	2.4 years		
		(5.08 %) (Dual-motor task)			
		(9.57 %) (Dual-cognitive task)			
*** Stride Length **% Decrease**	Kuhtz-Buschbeck et al. 2003 [9]	(13.21 %)	12.1 months	**(7 %)** (1-day post)	**(19 %)** (1-day post)
	Kuhtz-Buschbeck et al. 2003 [7]	(16 %)	2.8 months		
		(7.88 %)	7.8 months		
	Abdul-Rahman et al. 2021 [65]	(2.25 %) (Single task)	2.43 years		
		(6.92 %) (Concurrent motor task)		**(9 %)** (4-day post)	**(5 %)** (4-day post)
		(3.98 %)			

		(Concurrent cognitive task)			
	Howell et al. 2017 [67] (1 concussion)	(1.63 %) (Single task)	10.7 days		
		(0.93 %) (Double task)			
	Howell et al. 2017 [67] (⩾ 2 concussions)	(8.13 %) (Single task)	8.6 days	**(2 %)** (7-day post)	**(4 %)** (7-day post)
		(9.26 %) (Double task)			
	Berkner et al. 2017 [13]	(7.2 %) (Single task)	9.5 days		
		(7.3 %) (Double task)			
Cycle Time **% Increase**	Howell et al. 2017 [67] (1 concussion)	0.93 % (Single task)	10.7 days	**37 %** (1-day post)	**152 %** (1-day post)
		1.64 % (Double task)		**16 %** (4-day post)	**30 %** (4-day post)
	Howell et al. 2017 [67] (⩾ 2 concussions)	2.78 % (Single task)	8.6 days		
		6.56 % (Double task)		**18 %** (7-day post)	**30 %** (7-day post)
Stance Time **% Increase**	Karunakaran et al. 2020 [56]	107.5 %	2.4 years	**35 %** (1-day post) **19 %** (4-day post) **19 %** (7-day post)	**191 %** (1-day post) **33 %** (4-day post) **43 %** (7-day post)

*** Stride length represents average percent decrease for all legs (LF, LH, RF, RH).
